# Lipedema Research—Quo Vadis?

**DOI:** 10.3390/jpm13010098

**Published:** 2022-12-31

**Authors:** Anna M. Ernst, Hannelore Bauer, Hans-Christian Bauer, Marianne Steiner, Anna Malfertheiner, Anna-Theresa Lipp

**Affiliations:** 1 Department of Environment & Biodiversity, Paris Lodron University of Salzburg, 5020 Salzburg, Austria; 2 Institute for Tendon and Bone Regeneration, Paracelsus Medical University (PMU), 5020 Salzburg, Austria; 3 Department of Plastic Surgery and Hand Surgery, Klinikum rechts der Isar, Technical University of Munich (TUM), 81675 Munich, Germany; 4 Private Practice Dr. Lipp & Colleagues, 80331 Munich, Germany

**Keywords:** lipedema, adipose tissue, in vitro and ex vivo studies

## Abstract

When studying the current literature, one might get the impression that lipedema is a “modern” disease, with increasing incidence and augmenting prevalence throughout Western countries during the last decade. However, a quick look into older textbooks shows that disproportionate accumulation of fat in female bodies has long been known without being recognized as an independent disease. Nevertheless, it was not until 1940 that Allen and Hines described a *“syndrome characterized by fat legs and orthostatic edema”* in a seminal publication. The mere awareness that people who have lipedema are not just overweight but suffer from a yet poorly defined pathological condition, may be considered a decisive leap forward in the understanding of lipedema. A number of comprehensive publications have since dealt with the clinical presentation of lipedema and have provided the first clues towards the potential pathological mechanisms underlying its initiation and progression. Nevertheless, despite all effort that has been undertaken to unravel lipedema pathology, many questions have remained unanswered. What can be deduced with certainty from all experimental and medical evidence available so far is that lipedema is neither a cosmetic problem nor is it a problem of lifestyle but should be accepted as a serious disease with yet undetermined genetic background, which makes women’s lives unbearable from both a physical and psychological point of view. To date, results from clinical inspections have led to the categorization of various types and stages of lipedema, describing how the extremities are affected and evaluating its progression, as demonstrated by skin alterations, adipose tissue volume increase and physical and everyday-behavioral impediments. There is accumulating evidence showing that advanced stages of lipedema are usually accompanied by excessive weight or obesity. Thus, it is not unreasonable to assume that the progression of lipedema is largely driven by weight gain and the pathological alterations associated with it. Similarly, secondary lymphedema is frequently found in lipedema patients at advanced stages. Needless to say, both conditions considerably blur the clinical presentation of lipedema, making diagnosis difficult and scientific research challenging. The present literature review will focus on lipedema research, based on evidence fromex vivo and in vitro data, which has accumulated throughout the last few decades. We will also open the discussion as to whether the currently used categorization of lipedema stages is still sufficient and up-to-date for the accurate description of this enigmatic disease, whose name, strangely enough, does not match its pathologic correlate.

## 1. Introduction

### 1.1. Background

Lipedema (*adiposis dolorosa, lipomatosis dolorosa of the legs, lipoedema*) is a painful disorder characterized by the symmetrical accumulation of subcutaneous fatty tissue, particularly on the extremities (thighs, calves, upper arms) [1,2,3,4,5,6,7,8]. Lipedema affects predominantly, if not exclusively, women. Those cases in which lipedema was diagnosed in males were shown to be secondary to other diseases involving severe hormonal disturbances [9]. The worldwide prevalence of lipedema among women and post-pubertal girls is estimated at 11% [10]. However, due to a lack of accurate diagnostics markers and potential misdiagnosis, all statistical information must be viewed critically.

Diets and exercise as well as decongestive therapies (lymphatic drainage) and the wearing of compression garments may improve the patients’ overall condition, but cannot eliminate the pathological mechanism(s) underlying initiation and progression of the disease. Currently, most women choose liposuction as the most causative treatment, yielding long-lasting improvements [11,12,13,14,15,16,17].

It is generally agreed upon that lipedema manifests during periods of great hormonal fluctuations, usually during puberty and pregnancy, and in rare cases during menopause. A possible genetic predisposition appears plausible. A familial incidence of 15%, affecting first-degree female relatives, has been documented although self-reports usually tend to greatly exceed this percentage [9]. The mode of inheritance is considered to be either X-linked dominant or autosomal dominant with incomplete penetrance and sex limitation, but could also be oligogenic [9,18].

Currently, the visual inspection and an accurate survey of the patient’s medical history is basic to routine diagnosis of lipedema and may be improved by sonographic examination to distinguish lipedema from lymphedema at advanced stages of the disease [19,20,21]. An interesting approach to narrow down the diagnosis of lipedema was reported previously [22]. Thereby, the regional body composition, comprising fat mass, lean mass and bone mass in lipedema and non-lipedema women was measured using dual-energy X-ray absorptiometry. The authors reported that across all body mass index (BMI) categories, the fat mass of the legs related to the BMI was significantly higher in lipedema patients compared to healthy controls.

Today diagnosis of lipedema relies on criteria, originally established by Wold et al. in 1951 [23]. Following modifications introduced by Herbst [1], these criteria are still applied for routine diagnosis [7,24,25].

Based on the diagnostic criteria described so far, one might surmise that it should not be too difficult to distinguish lipedema from other phenotypically similar diseases, including obesity, lipohypertrophy or lymphatic and venous disturbances. In fact, the phenotypic appearance of normal weight patients is quite unambiguous, making an accurate diagnosis easy. Problems arise, when phenotypically similar clinical pictures overlap with lipedema [26]. The co-occurrence of lipedema and obesity is particularly frequent, which has even led to the notion that the progression of lipedema is driven by weight gain rather than by potential (yet undefined) lipedema-specific physiological alterations [27]. In this respect, the question arises, what “advanced lipedema” means, and how it could be studied without interference of overweight or obesity and obesity-associated pathologies.

The present literature review was conducted using PubMed to search for studies, which depict the progress and current status of research into lipedema. A special focus was placed on the results from ex vivo and in vitro studies.

### 1.2. “Lymph Makes You Fat”—Could This Be Relevant for Lipedema?

The question of whether lipedema is associated with lymphedema or not is all the more important as several lines of evidence have indicated that defective lymphatics may contribute to fat accumulation [28,29,30]. In this context, deletion of the transcription factor Prox-1 (prospero homeobox-1) in lymphatic endothelial cells in a mouse model led to the leakage of lymph, stimulating adipocytes to store fat and enhancing adult-onset of obesity [28]. Thus, a potential crosstalk between lymphatics and adipose tissue would be a welcome explanation for the locally restricted fat gain in lipedema.

It is still being debated as to whether vascular disorders are integral to lipedema, particularly at late stages. Since lipedema has originally been described as “a syndrome characterized by fat legs and orthostatic edema” [23], special attention has been paid to this important aspect from early on. Numerous imaging techniques have been used to study the lymphatic conditions of lipedema patients; however, with fairly inconsistent results. This may be partly explained by the frequently found overlap of lipedema and obesity within the patient cohorts studied.

While Bilancini et al. [31] reported a marked slowness of the lymphatic system in lipedema patients compared to healthy controls, only a moderate impairment of the lymphatic system in lipedema patients was found by Haarwood [32], emphasizing that the lymphatic function in lipedema was not comparably impaired as observed in lymphedema. A subclinical status of lymphedema in lipedema patients was also described by Lohrmann et al. [33] using magnetic resonance lymphangiography. In addition, during advanced stages of lipedema, multiple microlymphatic aneurysms of lymphatic capillaries have been described which might contribute to edema formation [34]. A morphological study using skin biopsies from lipedema thigh tissue revealed increased dermal spaces and abnormal vessel phenotype compared to controls, which could be an indication of a possible vascular disorder [35]. Further evidence supporting the notion of a lymphatic defect associated with lipedema came from Ma et al. [36], demonstrating elevated levels of PF4 (platelet-factor 4)/CXCL4) in circulating blood plasma exosomes from lipedema patients. According to these results, PF4 may be considered a novel biomarker for lymphatic defects unrelated to body weight.

On the other hand, considerable evidence has accumulated contradicting the assumption that lymphatic insufficiency is involved in lipedema etiology, as has been summarized in a recent publication [27]. For instance, magnetic resonance imaging (MRI) studies could not verify the presence of edema in subcutaneous fat and muscle tissue of lipedema patients [37]. Similarly, no dermal edema could be observed by high-resolution cutaneous ultrasonography in lipedema patients, focusing on the thigh or ankle region [19], and no edematous condition could be found in lipedema adipose tissue upon quantification of the local tissue water content [38].

In a recent study, dilated lymphatic vessels but no dermal backflow and no edema in lower extremities of lipedema patients at early stages was shown, suggesting that no lymphatic disturbance is involved in the etiology of early-stage (stage I and II) lipedema [39]. This further supports the notion that lymphedema may be induced by being overweight/obese which accompanies lipedema at advanced stages [40].

Taking together all medical evidence available so far, it is reasonable to suggest that edema formation due to lymphatic disorders appears to play only a minor, if any, role in the initiation of lipedema. During advanced stages of lipedema, venous or lymphatic insufficiency might exacerbate the clinical picture of lipedema. Whether or not these vascular disturbances are merely a consequence of weight gain needs to be clarified in more comprehensive systematic studies, with particular focus on the BMI and other weight-associated parameters of the participants.

Increased fragility of blood capillaries in lipedema adipose tissue was shown by Szolnoky et al. using angiosterrometry [41,42]. The authors demonstrated a substantial improvement of capillary fragility in lipedema patients, following complete decongestive therapy [42]. Morphological alterations of the microvasculature in lipedema adipose tissue microvessels of the lower papillary dermal layer were reported by Al-Ghadban et al. [43], showing increased capillary diameter in normal weight lipedema patients compared to controls. Lymphatic vessel areas did not differ between lipedema and non-lipedema adipose tissue from non-obese donors, but were significantly increased in adipose tissue from obese donors. No alterations of vessel morphology but increased systemic levels of VEGF-C in serum from lipedema patients and increased M2-polarized macrophage infiltration into lipedema adipose tissue were re-ported by Felmerer et al. [44], indicating a potential disturbance of the permeability properties in lipedema blood and lymphatic vasculature.

Interesting results from a recent study support the notion that the endothelial barrier is compromised in lipedema adipose vasculature [45]. The authors showed that soluble factors released from cultured lipedema SVF (stromal vascular fraction) cells led to a significant reduction of VE-cadherin (cadherin-5) expression in endothelial cells in an in vitro model. This effect could not be elicited by conditioned medium from control SVF cells, suggesting that the microenvironment of blood vessels in lipedema and non-lipedema adipose tissue differs substantially. VE-cadherin is an essential component of endothelial cell-to-cell adherens junctions and is critically involved in maintaining vascular permeability in addition to orchestrating numerous intracellular signaling pathways [46]. Thus, it may be speculated that a potential downregulation of VE-cadherin expression in the adipose tissue vasculature in vivo could lead to even more far-reaching effects than just alterations in permeability properties.

### 1.3. In Search of a Molecular Marker for Lipedema Diagnosis

There is still a risk that lipedema is being under- or over-diagnosed, particularly due to the above-mentioned co-occurrence of obesity, lymphedema and other pathological conditions that potentially mask lipedema. This has fueled a quest for the identification of unique, unambiguous molecular diagnostic markers of lipedema.

**Figure 1:**
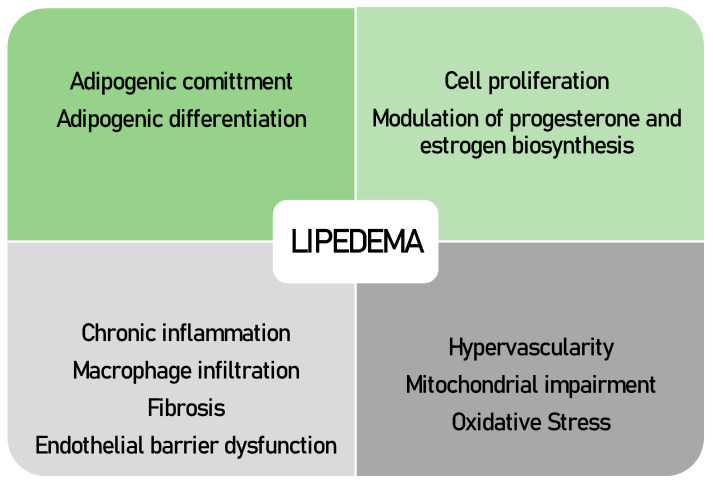
Biological processes suggested to be affected in lipedema pathology, as evidenced by data from ex vivo and in vitro research.

In this context, a number of studies have been conducted, applying histomorphology as well as cell-based molecular and immunological techniques (Table 1 and Table 2). Results from these studies show that lipedema manifests itself at many levels (Figure 1).

Particular attention has been paid to the characterization of a distinct population of adipose tissue resident mesenchymal stem cells, referred to as adipose tissue-derived stromal/stem cells (ASCs), from lipedema subcutaneous adipose tissue [60,61].

Since liposuction is currently the most causative treatment of lipedema, lipoaspirates from subcutaneous adipose tissue are available in sufficient quantity for research purposes, which may be considered a key benefit. Lipoaspirates are mainly used for isolation of the “stromal vascular fraction” (SVF), but also of mature adipocytes, residing in a different layer of the liposuction material. These cellular fractions may also be isolated from biopsied (solid) adipose tissues, though cell yield tends to be lower.

The SVF is a clinically interesting adipose tissue fraction, which is usually sedimented from the aqueous layer of enzymatically digested adipose tissue. It contains a heterogeneous population of cells, including endothelial cells and endothelial precursor cells, ASCs, pre-adipocytes, pericytes, smooth muscle cells, fibroblasts, lymphocytes and macrophages [62,63,64].

ASCs account for less than 0.1% of the SVF and are grown in 2D- or 3D cultures [65]. They are characterized by their growth behavior, multi-lineage differentiation potential and immunophenotypic markers [66,67,68,69,70]. ASCs may be kept in an undifferentiated state and may be expanded in vitro and serially passaged up to a limited passage number. Long-term expansion leads to alterations in morphology and proliferation kinetics [71]. These cells are easy to handle since they adhere well to the culture dish and do not show spontaneous differentiation (lipogenesis) when cultured in basic medium without adipogenic stimuli. In contrast, freshly isolated mature adipocytes float and would need to be kept in “ceiling cultures” [72].

### 1.4. Focusing on Adipogenesis

Adipogenic differentiation is a complex multi-step process that involves the stimulation of a transcriptional cascade and can be divided into two separate phases, adipogenic commitment and terminal differentiation [73,74,75]. Upon treatment with appropriate inductive media uptake of glucose is enhanced and adipogenesis/lipogenesis is initiated. Inductive media basically contain the glucocorticoid analog dexamethasone, insulin, indomethacin and the cAMP stabilizer IBMX (3-isobutyl-1-methyl-xanthine). Other optional other ingredients, leading to the augmentation of lipid droplet formation may be added [69,76,77].

The progression of adipogenic differentiation is usually evaluated bythe accumulation of lipid droplets (cytoplasmic triglycerides) and the corresponding alterations in adipogenic/lipogenic gene expression [78,79]. Lipid droplets (LD) are dynamic organelles, responsible for storage of free-fatty acids and neutral triglycerides. They also orchestrate the degradation of excess triglycerides by activating lipolysis and lipophagy [80].

**Figure 2:**
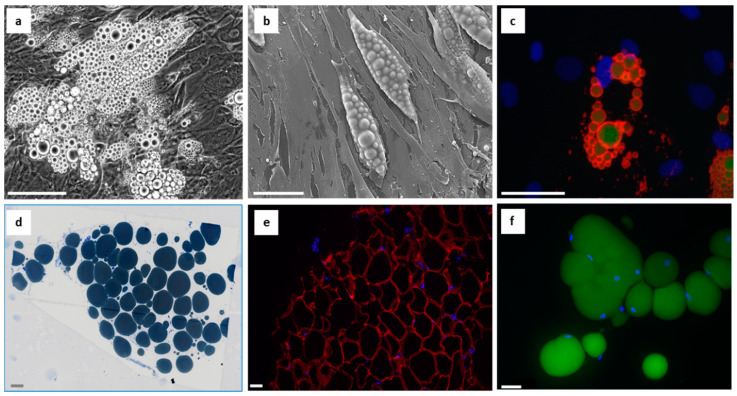
(**a**–**c**): In vitro differentiated adipocytes show multilocular lipid droplets after 21 days of adipogenic stimulation from (**a**) light microscopy (live cell imaging) (scale bar: 100 µm); (**b**) scanning electron microscopy (scale bar: 50 µm); (**c**) immunofluorescence staining of perilipin (red), LipidToxTM (green) and DAPI (blue) (scale bar: 50 µm). (**d**–**f**): Mature adipocytes in/from subcutaneous adipose tissue show unilocular lipid droplets from (**d**) semithin section from a resin-embedded biopsy-tissue, fixed with Karnovsky’s fixative and post-fixed with OsO4 stained with methylene blue/azurII; (**e**) immunofluorescence staining of a paraffin section from biopsied adipose tissue. Perilipin (red), DAPI (blue) (scale bar: 50 µm); (**f**) immunofluorescence staining of mature adipocytes isolated from liposuction material. LipidToxTM (green), DAPI (blue) (scale bar: 50 µm).

In vitro studies of lipedema adipose derived stem cells and preadipocytes aremainly performed in 2D cultures and in vitro differentiation of ASCs upon adipogenic stimulation is usually followed after 21–28 days of cultivation. Interestingly, in vitro differentiation, particularly in 2D cultures, leads to the accumulation of small multilocular lipid droplets, while freshly isolated mature adipocytes contain a single large unilocular LD [81] (Figure 2a–f).

So far, information concerning the biogenesis of lipid droplets in lipedema adipocytes is lacking but could contribute to the elucidation of the mechanisms underlying adipocyte hypertrophy in lipedema.

Only limited information exists concerning adipogenic gene expression in lipedema ASCs and adipocytes compared to healthy controls. A significant increase in expression of the master transcriptional regulator PPARγ (peroxisome proliferator-activated receptor gamma) and a higher adipogenic potential of lipedema ASCs compared to controls was reported by Al-Ghadban et al. using 2D cultures [55]. This is partly supported by data showing significant upregulation of various adipogenic genes, including PPARγ, CD36/fatty acid translocase, and the fatty acid binding protein-4 (FABP4) in in vitro differentiated lipedema adipocytes from non-obese donors compared to non-lipedema controls [51]. In contrast, other studies did not reveal significantly increased expression of adipogenesis-related genes in lipedema adipose cells [45,48]. A significant upregulation of the transcription factor ZNF423 (Zfp423) in lipedema SVF cells compared to controls was reported recently [45]. Since ZNF423 is critically involved in early adipocyte commitment [82], a potential dysregulation of lipedema adipose tissue formation at an early stage of mesenchymal stem cell differentiation is possible.

### 1.5. Focusing on Hypertrophy and Hyperplasia

Accumulation of adipose tissue in vivo relies principally on hyperplasia (cell number increase) or hypertrophy (cell size increase) of adipocytes [83]. The hyperplastic expansion is accomplished by the constant replenishment of ASCs and preadipocytes, which differentiate into mature adipocytes upon environmental cues. From a metabolic point of view, hyperplastic expansion is considered more favorable for the organism compared to hypertrophy. The latter occurs in response to excessive accumulation of triglycerides, which may also be triggered by a disturbed balance of lipogenesis and lipolysis [84]. Without having a sufficiently accurate picture based on scientific data, it appears plausible that both hyperplasia and hypertrophy contribute to the fat accumulation in lipedema adipose tissue, although the initial triggering signal is unknown.

A significant increase in adipocyte size of adipose tissue from non-obese lipedema donors compared to non-obese controls was reported recently [43]. Interestingly, adipocyte areas did not differ between samples from obese lipedema patients compared to obese controls, suggesting that it is a sine qua non to study lipedema-specific characteristics exclusively in non-obese patients. Adipocyte hypertrophy and an aberrant lipid metabolism in lipedema adipose tissue was reported by Felmerer et al. [48]. Accordingly, hypertrophy of lipedema adipocytes in paraffin-embedded adipose tissue compared to controls was also observed by Wolf et al. [49].

Hyperproliferation of cultured lipedema ASCs was reported by Bauer et al., describing a proliferative boost after a lag period of comparable growth between lipedema and non-lipedema ASCs [54]. Accelerated proliferation of lipedema ASCs compared to controls was also observed by Musarat Ishaq [50]. Moreover, the authors demonstrated increased expression of Bub1, a cell-cycle regulator involved in cell proliferation and increased activation of histone H2A, in lipedema ASCs. A significantly elevated SVF cell yield isolated from lipoaspirates from lipedema patients compared to non-lipedema controls was also reported by Priglinger et al., [53], though cell doubling time did not differ between lipedema and non-lipedema ASCs in culture. Increased expression of CCND1/cyclin D1, a cell cycle regulator that is usually increased upon proliferative signals, was detected in lipedema but not control adipocytes [48]. This finding further supports the notion of a potential hyperproliferative activity of lipedema ASCs.

### 1.6. Chronic Inflammation and Oxidative Stress—Primary or Secondary to Lipedema?

Adipose tissue hypertrophy is usually associated with increased cell death and the concomitant invasion of macrophages scavenging adipocyte debris. Macrophages engulfing dying adipocytes are histologically presented as “crown-like structures” and are actually considered a pathologic hallmark of obesity [85]. Although crown-like structures have often been associated with lipedema, there is more evidence for assigning this histological finding to obesity rather than to lipedema.

In addtion to increased macrophage infiltration, upregulation of inflammatory genes and proteins in lipedema adipose cells and tissue has also been reported [48,54]. A significant upregulation of IL-8 levels in supernatants of cultured lipedema ASCs compared to controls was observed by Bauer et al. [54], suggesting that ASC-derived proinflammatory cytokines may promote the local inflammatory milieu in lipedema adipose tissue. Upon adipogenic induction, IL-8 levels dropped to equally high basal levels in lipedema and non-lipedema adipocytes [54]. Serum samples from lipedema patients exhibited increased levels of IL-28A and -29 and the adipocyte-derived IL-11 [49]. Liposuction led to a significant reduction of systemic INFα2 and IL-34 [49], the latter being associated with obesity-related inflammation and other obesity-related pathologies [86]. A trend of elevated expression of VEGF, IL-6, IL-1ß, and TNFα in differentiated lipedema adipocytes compared to controls was observed by Al-Ghadban [55], principally in adipose tissue derived from the thigh but not abdomen of donors. A significant decrease of IL-8 but not IL-6, IL-1ß or TNFα was found in supernatants from lipedema SVFs [45]. It is particularly important to emphasize that inflammatory cytokines play a critical role in endothelial-barrier function [87,88,89,90,91] and thus may be important players in the initiation and progression of lipedema.

Particularly limited information exists concerning the assumption that lipedema is caused by or accompanied by mitochondrial disturbances. Using a mitochondrial stress test, a significant increase of the maximal possible oxygen consumption after stimulation with the uncoupler FCCP of lipedema SVF cells compared to controls was found [49]. Basal mitochondrial respiration did not differ between lipedema and control cells. This supports earlier findings suggesting mitochondrial dysfunction and oxidative stress in lipedema adipose tissue. The authors reported increased serum concentrations of malondialdehyde and plasma protein carbonyls compared with healthy control persons [52].

## 2. Future Considerations

There is no doubt that experienced physicians are able to correctly diagnose lipedema in daily clinical routine. On a scientific basis, however, the clarity to diagnose lipedema upon specific “markers” is currently not given, be it on a histological, cell biological or molecular level.

A recent publication states that facts and myths about lipedema are often confused and passed on in a kind of tradition [27]. This is even more astonishing since it would be very easy to verify assumptions and claims with scientific evidence or to prove them wrong.

As summarized above, considerable scientific data based on ex vivo and in vitro studies have accumulated during the last couple of years. Most of these studies focused on various aspects of adipose tissue differentiation and the cellular and molecular microenvironment of adipose cells in vivo and in vitro. Why has so little success been achieved in the case of lipedema?

In the following, some possible explanations are given, and an attempt is made to provide an improved approach for future studies. Knowledge, which has accumulated during the last few years, should be harnessed to develop optimized strategies for ex vivo and in vitro study on lipedema, which can then represent a real and important complement to the daily clinical routine.

### 2.1. The Issue of Patients’ Weight and the Dilemma of Assembling a Representative Cohort

It is generally agreed upon that obesity/weight gain accompanies lipedema particularly during advanced stages. Thus, a normal weight patient exhibiting lipedema stage 3 is very rare. The most serious mistake found in various reports of cohort studies is the high BMI range within the observed groups/cohorts.

It is known that the influence of weight, in particular obesity, is decisive for many histological and cell-biological properties. Thus, the interpretation of data emerging from studies with a donor population whose BMI ranges from normal weight or overweight to obesity, becomes difficult, if not impossible.

In addition, it is rightly being debated as to whether the BMI is a suitable tool to evaluate the body mass of lipedema patients. Due to the strong inequality of the upper and lower body, the indication of the total weight can be misleading. It would be advisable to use both the BMI and the hip/waist ratio to estimate and categorize the donor cohorts.

### 2.2. The Dilemma with Edema and Vascular Disturbances/Dysfunctions

As previously mentioned, this topic has extensively been discussed, probably due to the phenotypically similar picture of lymphedema and advanced lipedema stages. However, it is striking that the data from the literature are contradictory. Why are data concerning a prospective involvement of vascular disturbances in lipedema pathology so inconsistent? Why could the question concerning a potential connection of vascular dysfunction in lipedema not be answered satisfactorily for so long?

Here, too, the reason seems to lie in the poor selection of the subjects. Again, numerous studies concerning vascular conditions of lipedema patients were performed with patients exhibiting excessive weight (obesity) or with cohorts comprising a mixture of normal weight and obese patients.

It cannot be stressed enough that any overlap of lipedema and obesity is an enormous obstacle in generating a clear picture of the vascular conditions of lipedema patients and to identify lipedema-specific alterations.

Further, it cannot be entirely ruled out that lipedema adipose tissue could harbor microedema, which is undetectable by standard methods and can only be seen with high-resolution ultrastructural imaging. It would therefore be advisable to significantly expand the range of methods for the histological examination of the lipedema adipose and vascular tissue to include electron microscopic analyses.

### 2.3. The Dilemma with Age and Interdonor Variability

When reviewing the literature, it is noticeable that patient cohorts with a large age range are included in many studies. Since the development and progression of lipedema is obviously triggered by hormonal conditions, it would be advantageous to narrow down the age range of study cohorts to ensure a comparable hormonal status of participants as this is crucial for the development of adipose tissue.

This could also have a positive effect in reducing the high interdonor variability, which very often leads to valueless data because they are outside statistical significance.

### 2.4. The Dilemma with “Controls”

Most of the ex vivo and in vitro studies include age- and BMI-matched controls in order to pinpoint lipedema-specific characteristics. It is reasonable to assume that people undergoing cosmetic liposuction suffer from excessive localized fat accumulation, whether due to overweight/obesity or lipohypertrophy. Neither condition is an optimal control for lipedema. This is all the more important to reconsider since it cannot be excluded that lipohypertrophy and lipedema share similar molecular mechanisms underlying adipose tissue accumulation. These considerations are of secondary importance for the surgeon but play a crucial role when it comes to evaluating and interpreting scientific data.

### 2.5. Is the Current Classification in Light of the Data and Experience Collected from Recent Years Still Sufficient?

Since increasing medical and scientific interest has been focused on lipedema during the last couple of years, many observations have been made which would eventually justify a revision of the current staging of lipedema. Each lipedema stage must be seen as a complex entity since it represents the sum of several conditions. Thus, it would be advisable to extend the current staging by indicating additional information such as the occurrence of co-morbidities (obesity, lymphedema, and others), the patient’s original weight (in case of multiple operations), and the family history. A more differentiated classification would correspond to the heterogeneous presentation of this clinical picture.

## 3. Summary

In recent years, lipedema research has evolved from purely clinical testing to molecular science. In this short review, we have tried to give an overview of the diverse laboratory-technical approaches and the findings obtained from them. Excellent and time-consuming procedures have shown that lipedema manifests itself on many levels and have once again confirmed that in vitro and ex vivo studies are essential to understand the multifaceted nature of the disease.

However, what has also become clear is that many questions are still unanswered, and a lot of preliminary data still needs to be deepened. We also addressed the main obstacles hampering lipedema research. Currently, there is no mouse model, no cell lines which could be used to achieve reproducible results. Therefore, one of the biggest handicaps, the high interdonor variability, that often makes scientific results unusable, most likely will persist. It is also evident that too little attention has been paid to the problem of accompanying obesity for too long. This does not mean that we have to start from scratch, but we need to start discarding some unverifiable opinions about lipedema. As outlined above, there are already valuable indications regarding in which direction(s) lipedema research should develop. Implementing new findings and merging data from different disciplines will pave the way to the successful elucidation of this enigmatic disease.

## Figures and Tables

**Table 1:** Histological/morphological, histochemical and immunocytochemical studies.

METHODS	TISSUE/CELLS	RESULTS	AUTHORS
Immunostaining	Paraffin-embedded adipose tissue Histological sections	Increased infiltration of CD68+ macrophages in lipedema adipose tissue and occurrence of “crown-like structures”; large number of KI67^+^/CD34^+^ proliferating cells in lipedema tissue	[47]
Histochemical staining Immunostaining	Paraffin-embedded adipose tissue Histological sections	Hypertrophy of adipocytes in adipose tissue from non-obese lipedema donors; increased macrophage density in lipedema skin and fat; increased numbers of dermal blood vessels in lipedema adipose tissue; increased dilatation of capillaries in non-obese lipedema adipose tissue compared to non-obese controls	[43]
Immunostaining	Paraffin-embedded adipose tissue Histological sections	Increased M2-macrophage infiltration into lipedema adipose tissue; no morphological changes in lymphatic and blood vasculature; no changes in number, size or percentage coverage of lymphatic vessels or blood vessels in lipedema tissue sections	[44]
Histochemical staining Immunostaining	Paraffin-embedded adipose tissue Histological sections	Increased dermal spaces and abnormal vessel phenotype (rounded endothelial cells; perivascular spaces, perivascular immune cell infiltrate) in lipedema specimens compared to controls	[35]
Histochemical staining Immunostaining	Paraffin-embedded adipose tissue Histological sections	Increased epidermal thickness in lipedema patients; adipocyte hypertrophy, increased fibrosis and significant increase in CD68+ macrophages in lipedema tissue	[48]
Histochemical staining Immunostaining	Paraffin-embedded adipose tissue Histological sections	Significant hypertrophy of lipedema adipocytes	[49]
Immunohistochemistry BODIPY staining of droplets	Paraffin-embedded adipose tissue Histological sections	Significantly increased number of CD29/CD34 positive cells in lipedema adipose tissue; enhanced adipogenic potential of lipedema ASCs	[50]
Histochemical staining Immunostaining Machine learning analysis	Paraffin-embedded adipose tissue Histological sections SVF, ASCs	No difference in epidermal thickness of thigh tissue between lipedema and control tissue; no signs of fibrosis; no alterations in lymphatic endothelial cells in lipedema adipose tissue; higher number of CD68+ macrophages in CD31+/podoplanin- areas of lipedema tissue; morphological alterations of interendo-thelial junctions between lipedema en-dothelial cells in vitro	[45]
Immunostaining Histochemical staining	SVF, ASCs In vitro differentiated adipocytes	Increased occurrence of myofibroblast-like cells in lipedema adipocytes from normal weight and overweight lipedema donors	[51]

**Table 2:** Molecular studies and cell-based functional assays.

METHODS	TISSUE/CELLS	RESULTS	AUTHORS
HPLC	Blood samples Plasma	Increased parameters of oxidative stress (plasma MDA and plasma protein carbonyl concentrations) in lipedema patients compared to controls	[52]
Immunophenotyping Flow cytometry OilRed O staining	Lipoaspirates SVF/ASCs In vitro differentiated adipocytes	Enhanced SVF cell yield in lipedema preparations with increased CD90 and CD146-positive cells; reduced in vitro differentiation capacity of lipedema ASCs	[53]
ELISA OilRedO staining Cell counting	Lipoaspirates SVF/ASCs In vitro differentiated adipocytes	Increased proliferative activity of lipedema ASCs; increased IL-8 levels in supernatants from lipedema ASCs; reduced adipokine and aromatase levels in supernatants from in vitro differentiated lipedema adipocytes; reduced differentiation capacity of lipedema ASCs	[54]
Proliferation assay CFU fibroblast assay qPCR OilRedO staining	Lipoaspirates SVF/ASCs (2D cultures) In vitro differentiated adipocytes	Significant increase in CFU potential and higher adipogenic potential of lipedema ASCs; increased expression of leptin and PPARγ in lipedema adipocytes; no change in proliferation rate of lipedema ASCs compared to controls; comparable inflammatory gene expression in lipedema and control ASCs and adipocytes	[55]
qPCR OilRedO staining	ASCs spheroids (3D cultures)	No difference in adipogenic gene expression (ADIPOQ, LPL, PPARγ, Glut4) between lipedema and healthy 3D-differentiated adipocytes; upregulation of IL-6 expression in 3D cultures of lipedema ASCs and adipocytes; elevated CFU activity and adipogenic potential of ASCs grown as spheroids	[56]
qPCR ELISA	Adipose tissue Serum	Increased levels of VEGF-C in serum from lipedema patients; increased expression of VEGFR-3 in lipedema adipose tissue; significant decrease in VEGF-A and VEGF-D, and Tie-2 expression in lipedema adipose tissue	[44]
Gene array of adipose-tissue related genes ELISA	Adipose tissue Serum	Aberrant lipid metabolic profile, increased cholesterol, triglycerides and LDL and ApoB in lipedema serum; no alteration in cytokine profile (IL-6, IL-18, lipocalin-2 and leptin); upregulation of CCND1/cyclinD1 and downregulation of CEBP, CFD, NCOR2, KLF4 in lipedema adipocytes	[48]
Analysis of extracellular miRNAs from SVF	Lipoaspirates; conditioned medium from SVF cells; small extracellular vesicles (sEVs)	Identification of lipedema-relevant miRNAs preferentially in sEVs; potential involvement of differentially expressed miRNAs in Notch, Wnt SMAD/TGFß-pathway, oxidative stress and senescence	[57]
Mass spectrometry analysis	Blood plasma exosomes (mouse and human)	Increase platelet factor 4 (PF4) levels in circulating exosomes from patients with lipedema	[36]
Whole exome sequencing qPCR Molecular modeling	Blood samples (germline DNA)	Discovery of a missense variant in the AKR1C1 gene encoding an aldo-keto reductase involved in progesterone metabolism	[58]
Lipidomic analysis (lipid mass spectrometry) Cytokine profiling (Multiplex immunoassay) Mitochondrial stress test	Adipose tissue biopsies Lipoaspirates SVF Serum	Significant increase in IL-11, IL-28A, IL29 expression in lipedema serum; no significant alteration in lipid composition in adipose tissue and serum from lipedema donors; significantly increased oxidative metabolism (enhanced mitochondrial function) of lipedema SVF cells	[49]
Transcriptional profiling Lipidomic and metabolomic analyses Functional assays BODIPY staining	Whole adipose tissue biopsies ASCs In vitro differentiated adipocytes	Differential expression of >4400 genes partly involved in cell cycle/cell proliferation and lipid metabolism, in lipedema adipose tissue, >900 changes in lipid composition and >600 differentially altered metabolites in lipedema adipocytes: differential expression of >3400 genes, partly involved in extracellular matrix, cell-cycle/proliferation signaling pathways, in lipedema ASCs; upregulation of the cell cycle regulator Bub1 and enhanced activation of histone H2A in lipedema ASCs; enhanced proliferation and differentiation of lipedema ASCs	[50]
qPCR Protein array Endothelial permeability assay	Lipoaspirates Whole adipose tissue (AT) SVF human primary ECs (hECs) SVF-derived sorted EC/PC SVF cell-derived conditioned medium (CM)	Significantly increased ZNF423 in lipedema SVF, EC and PC compared to controls; significant upregulation of aromatase expression in lipedema whole adipose tissue; lipedema SVF cell-induced dysfunction of the vascular endothelial barrier in vitro	[45]
RT-PCR, qPCR	Lipoaspirates ASCs In vitro differentiated adipocytes	Significant upregulation of PPARγ, CD36 and FABP4 in differentiated adipocytes from non-obese lipedema donors; reduced adiponectin/leptin ratio in obese but not non-obese lipedema adipocytes	[51]
Next-generation sequencing; multi-gene panel	Genomic DNA from peripheral blood	Identification of 21 deleterious variants in genes linked to syndromic fat accumulation (ALDH18A1, GHR) and differential diagnosis (PLIN1, LIPE, PPARγ, POMC, NR0B2, GCKR, NPC1), as well as lipedema candidate genes (RYR1, PPARA)	[59]

## Data Availability

No new data were created or analyzed in this study. Data sharing is not applicable to this article.
